# Supplementary material for: A decay effect of the growth rate associated with genome reduction in Escherichia coli
Source: BMC Microbiol. 2018 Sep 3;18:101. doi: 10.1186/s12866-018-1242-4 (PMC6122737; doi:10.1186/s12866-018-1242-4)
Supplement: Supplementary file 1 — Supplemental Figures S1-S5. (PDF 1767 kb) [file 12866_2018_1242_MOESM1_ESM.pdf]

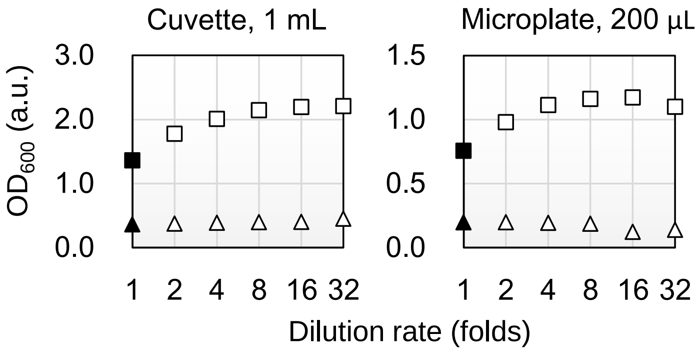

Figure S1 Mechanical errors in the method of optical turbidity. *E. coli* cells grown until either a high (closed squares) or low (closed triangles) density were measured by means of optical turbidity using both the cuvette (left panel) and the microplate (right panel). The cell cultures (closed triangles and squares) were subsequently and repeatedly subjected to a twofold dilution and measured at OD_{600}. The true OD_{600} values were calculated by multiplying the OD_{600} read of the diluted culture and its dilution rate (open triangles and squares). The changes in the true OD_{600} values were detected in the cultures of high but not low density. The mechanical errors of the optical measurements could be ignored in the evaluation of the growth rate, which is estimated according to the OD_{600} reads with a low density.

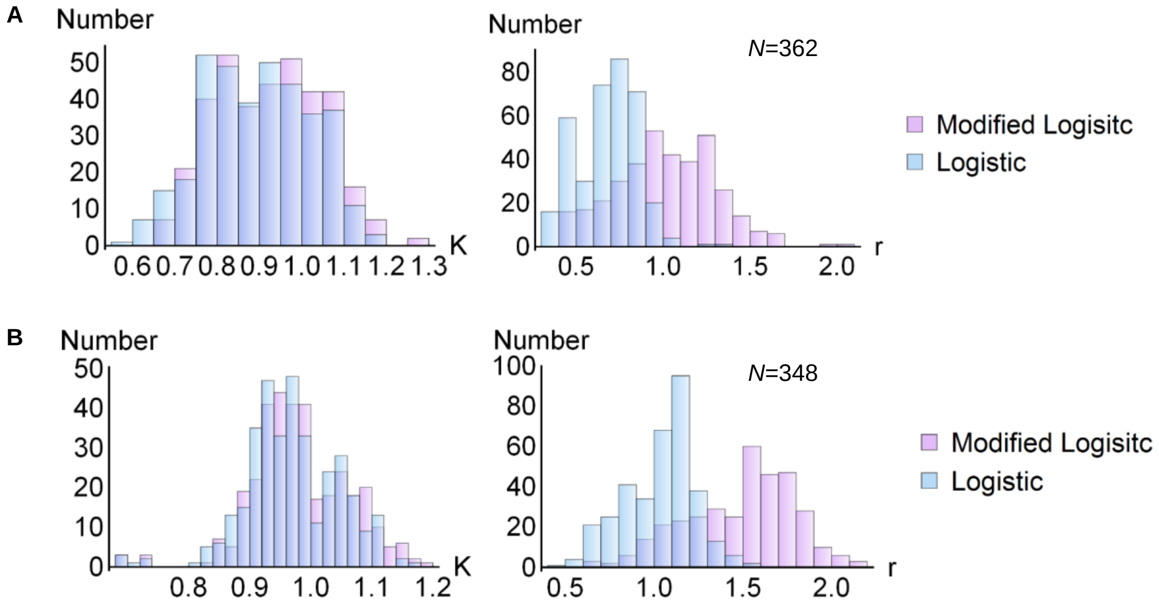

Figure S2 Histograms of *K* and *r* estimated by the two models. A. Histograms of *K* and *r* estimated by the two models in M63. **B.** Histograms of *K* and *r* estimated by the two models in LB. A total of 362 and 348 growth curves of 29 *E. coli* strains acquired in the media of M63 and LB, respectively, were subjected to the fitting (estimation). Blue and purple transparent colors stand for the fitting with the logistic and modified logistic models, respectively. The left and right panels indicate the parameters of *K* and *r*, respectively.

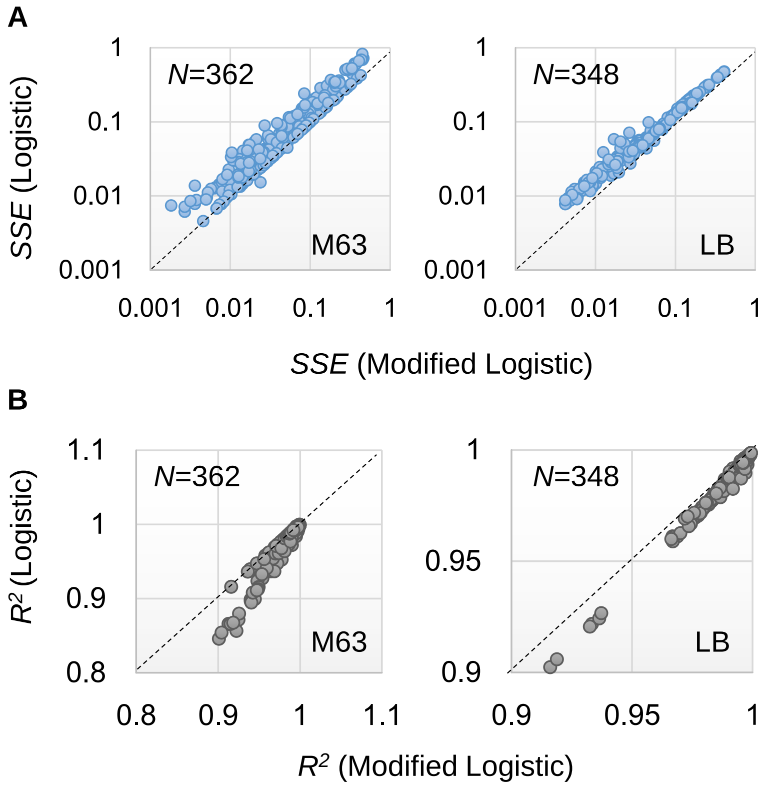

Figure S3 Fitting efficiency of the two models in M63 and LB. A. A comparison of the fitting of the residual errors between the two models. The fitting errors are represented by the sum of squares error, *SSE*. **B.** A comparison of the goodness of fit between the two models. The goodness of fit was represented by the coefficient of determination, R^2. A total of 362 and 348 growth curves of 29 *E. coli* strains acquired in M63 and LB media, respectively, were subject to the fitting. The broken lines indicate the equivalent fitting efficiency of the two models.

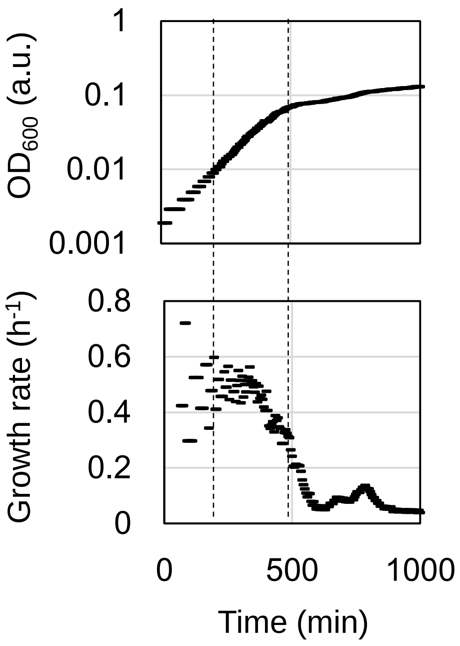

Figure S4 Decrease in growth rates during the exponential growth phase. Growth of *E. coli* cells growing in M63 were recorded at intervals of 5 min using a 96-well microplate (upper panel). Growth rates at every 1 h (20 intervals) were calculated as described in the Materials and Methods. Temporal changes of the growth rates in 5 min slices are shown (bottom panel). The growth rates were inconsistent even during the exponential growth phase, which is indicated by the broken lines.

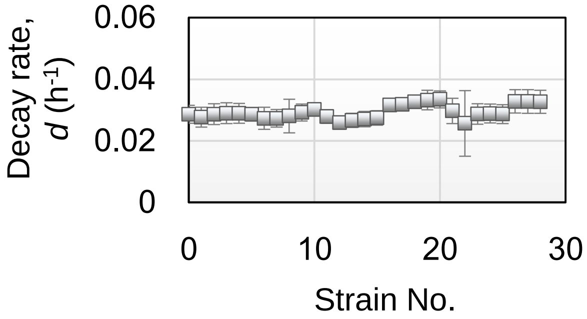

Figure S5 Relationship between the genome reduction and decay rate in LB. The mean decay rates results from the fitting of the growth curves in LB with the modified logistic model are shown in the order of the strain No. The genomes of strain Nos. 1~28 stand for the reduced genomes, and No. 0 indicates the wild-type genome W3110. The standard errors of the growth curves/tests (N=12) of the same strain are shown.
